# Comparative Flight Activities and Pathogen Load of Two Stocks of Honey Bees Reared in Gamma-Irradiated Combs

**DOI:** 10.3390/insects8040127

**Published:** 2017-11-29

**Authors:** Lilia I. de Guzman, Amanda M. Frake, Michael Simone-Finstrom

**Affiliations:** Honey Bee Breeding, Genetics and Physiology Laboratory, USDA-ARS, Baton Rouge, LA 70820, USA; mandy.frake@ars.usda.gov (A.M.F.); michael.simonefinstrom@ars.usda.gov (M.S.-F.)

**Keywords:** gamma irradiation, flight activities, foraging behavior, Russian honey bees, radio frequency identification

## Abstract

Gamma irradiation is known to inactivate various pathogens that negatively affect honey bee health. Bee pathogens, such as *Deformed wing virus* (DWV) and *Nosema* spp., have a deleterious impact on foraging activities and bee survival, and have been detected in combs. In this study, we assessed the effects of gamma irradiation on the flight activities, pathogen load, and survival of two honey bee stocks that were reared in irradiated and non-irradiated combs. Overall, bee genotype influenced the average number of daily flights, the total number of foraging flights, and total flight duration, in which the Russian honey bees outperformed the Italian honey bees. Exposing combs to gamma irradiation only affected the age at first flight, with worker bees that were reared in non-irradiated combs foraging prematurely compared to those reared in irradiated combs. Precocious foraging may be associated with the higher levels of DWV in bees reared in non-irradiated combs and also with the lower amount of pollen stores in colonies that used non-irradiated combs. These data suggest that gamma irradiation of combs can help minimize the negative impact of DWV in honey bees. Since colonies with irradiated combs stored more pollen than those with non-irradiated combs, crop pollination efficiency may be further improved when mite-resistant stocks are used, since they performed more flights and had longer flight durations.

## 1. Introduction

The performance and productivity of honey bee colonies greatly depends upon their health. Worldwide, the continued decline in honey bee health impedes the profitability of beekeeping operations. Among the many reported colony stressors, parasitic mites and their associated pathogens play major roles in colony health decline [1], both directly and indirectly. The ectoparasitic mite *Varroa destructor* (hereafter referred to as varroa mites) not only causes the death of infested hosts, but also alters the non-associative learning ability, flight duration, and homing ability of honey bee foragers [2,3,4]. Various pathogens also threaten honey bees’ health. *Deformed wing virus* (DWV), which is characterized by causing wing deformities in honey bees, is transmitted vertically from queen to worker, horizontally from worker to worker or worker to larvae, and by varroa mite-feeding [5]. Importantly, the mode of transmission, whether oral from worker to worker transmission or injection from varroa mite-feeding, highly influences the development of lethal symptoms resulting from DWV infection [6,7]. In addition to varroa mite-feeding as a route of pathogen infection for honey bees, diseases can also be acquired from used combs since the wax harbors many contaminants, including various pathogens. Some of the pathogens that cause bee diseases, such as American foulbrood (AFB) [8] and chalkbrood [8], are known to linger in honey bee combs and maintain infectivity. At present, the burning of infected bees and equipment is the only control measure to prevent the spread of AFB [9]. In addition, various micro-organisms have been isolated from the feces of honey bee larvae [10], and so this could also be a pathogen reservoir. For example, one of the main modes of transmission of the microsporidian gut parasites, *Nosema* spp., is through the cleaning of adult honey bee fecal matter that is deposited in and around the hive [11]. Chronic bee paralysis virus (CBPV) has also been detected in feces of infected adult bees [12]. Since varroa mites usually deposit their fecal matter on cell walls [13], this material may remain on cell walls or in between cocoon layers, a common observation in cells previously infested with *Tropilaelaps* spp. (LIG personal observation). Thus, it is possible that mite feces may also be infected with bee viruses and act as a reservoir for infection through the contamination of larval food. Like varroa mites, *Nosema* and viruses also have strong deleterious effects on honey bee foraging and survival [14,15,16,17,18,19].

The best way to eliminate pathogens is to prevent contamination. Gamma irradiation technology has been used to reduce exposure to and control pest problems in a variety of settings. It is currently used in food decontamination to kill food-borne pathogens and pests to increase the shelf life of various fruits and vegetables [20,21]. It has also been used to sanitize beekeeping equipment [22] and kill AFB spores [23,24,25,26]. Gamma irradiation has also been demonstrated to inactivate various viruses [27,28] and *Nosema apis* cysts [29]. Recently, it has been demonstrated to fully inactivate DWV, *Ascosphaera apis*, and *Nosema ceranae*, while only partially inactivating *Black queen cell virus* (BQCV) and CBPV (Simone-Finstrom et al. submitted). However, gamma irradiation of combs has been shown to be ineffective against *Streptococcus pluton*, the causative agent of European foulbrood [30].

The resistance to diseases or parasitic mites among stocks of honey bees varies [31,32,33,34]. Likewise, several components of foraging behavior are known to be varied based on bee genotypes both within and across colonies [35,36,37,38]. In this study, we determined whether or not worker bees reared in combs that were gamma irradiated are healthier (free from or less exposed to pathogens), and thus perform more flight activities than bees reared in non-irradiated combs. We also asked the question of whether or not the flight activities of varroa-resistant bees differ from those of a varroa-susceptible stock. We used flight activity as a proxy for colony performance and fitness [39].

## 2. Materials and Methods

### 2.1. Establishing Test Colonies

Forty colonies (1.4 kg) of bees were established from two large packages in order to obtain a uniform mixture of bees and varroa mites. Colonies were housed in 10-frame medium boxes, and each received one of the following four treatments: (1) Italian honey bee queen with irradiated combs (10 colonies); (2) Italian honey bee queen with non-irradiated combs (Control, 10 colonies); (3) Russian honey bee queen with irradiated combs (10 colonies); and (4) Russian honey bee queen with non-irradiated combs (Control, 10 colonies). The test combs were a mixture of old (had been previously used for brood rearing) and new (never used for brood rearing) combs. The irradiated combs and beekeeping equipment were exposed to 25 KGy using a cobalt-60 gamma irradiator (Food Technology Service, Inc., Mulberry, FL, USA). The Italian queens were purchased from a queen breeder that advertised Italian queens, while the Russians queens were produced from the Russian honey bee program [40,41]. 

### 2.2. Monitoring Flight Activities

Radio Frequency Identification (RFID) technology has been used to understand the foraging activities of bumble bees and ants [42,43] and to understand the effects of stressors in the context of foraging behavior in honey bees [16,18,39,44,45,46,47]. The RFID system used in this study was a MAJA Bundle: iID 2000, 13.56 MHz, ISO 15693 optimized, which consisted of an iID^®^ MAJA reader module 4.1, an iID^®^ HOST type MAJA 4.1, and a mic3^®^ RFID transponder (Microsensys GmbH, Efurt, Germany). Two readers were attached to the entrance of the host colony that recorded the times a tagged bee left and returned to the host colony.

### 2.3. RFID-Tagging and Paint-Marking of Bees

When the first brood laid on the test combs was about to emerge, the brood frames were removed and placed in an incubator. The next day, emerging bees were weighed and either RFID-tagged (*n* = 50 per colony) or paint-marked on the thorax with Testors enamel paint (*n* = 100 per colony). The two different types of marking were used as a way to determine if the RFID-tags reduced the survival of bees, and so paint-marking simply served as a control regarding lifespan effects. To ensure the mite infestation status of each test bee, bees were collected as they chewed their way out or started chewing the cell capping. Only bees that were not infested with varroa mites during their development were used in this study. All bees were weighed prior to RFID-tagging or paint-marking. Using a pair of forceps, newly emerged bees were tagged using a small amount of Loctite super glue applied on a bee’s thorax. RFID-tagged and paint-marked bees were placed in separate containers with sponges moistened with sucrose solution to allow time for the paint to dry and the bees to recover. Both RFID-tagged and paint-marked bees were introduced around dusk into two separate host colonies using push-in cages (eight-mesh screen; length = 20 cm, width = 16 cm, height = 2.5 cm) on a brood frame with some nectar or honey to increase acceptance. The bees were released the following day. Bees that died within the cage were counted and excluded from the total number of bees introduced.

Due to the large number of bees and colonies tested, not all bees were RFID-tagged or paint-marked on the same days. Bees from colonies that were tested on the same day are referred to as a “cohort” because temporal effects can often cause differences in behavior. Cohorts of bees were tagged or paint-marked on 19 May 2015 (*n* = 8; two colonies each for all treatment groups), 21 May 2015 (*n* = 8; two colonies each for all treatment groups), 26 May 2015 (*n* = 8; two colonies each for all treatment groups), and on 28 May 2015 (*n* = 6; one colony each for the non-irradiated groups and two colonies each for the irradiated groups). A total of 30 colonies (15 Russian, 15 Italian) were used as sources of test bees assessed in this study (see Table 1). Flight activities were monitored from 21 May 2015 to 5 July 2015 or until no more flights were recorded.

### 2.4. Host Colonies for RFID-Tagged and Paint-Marked Bees

In order to provide similar environmental pressure for all of the test bees, “host colonies” were used. Honey bees are known to adjust their activities according to the needs of the colonies [48]. By using a host colony, our test bees should have responded to the same colony needs. Hence, we are more confident that any differences in foraging activities observed are due to either bee genotype or comb treatment and not due to colony differences. One host colony each for the tagged and painted bees, which comprised one deep box (six brood frames and three food frames), one medium box with six food frames (nectar and pollen), and four empty frames, were used. The host colony for the RFID-tagged bees was headed by a naturally mated supersedure queen of Italian ancestry, while the host colony for the paint-marked bees had a Russian queen. Both host colonies were separate from the test colonies. A bee was considered dead if no reads were detected for her tag. For the paint-marked bees, survival was determined by a weekly census of all bees in the host colony. Assessments were conducted between 10:00 and 11:00 a.m. We used a different host colony for the paint-marked bees to avoid a disruption of the flight recordings of RFID-tagged bees due to a prolonged weekly census.

### 2.5. Viral Load, Nosema Spore Count, and Amount of Pollen Stores

For the viral load analysis, eight newly emerged bees from each of eight colonies (irradiated = 4 colonies; non-irradiated = 4 colonies) were subsampled prior to RFID-tagging in May and then again in June and October. The RNA extractions were completed using the Maxwell^®^ 16 LEV simplyRNA system (Promega, Madison, WI, USA), and cDNA template was generated from 2 µg of total RNA using the QuantiTect Reverse Transcription Kit (Qiagen, Germantown, MD, USA) following the manufacturer’s instructions. qPCR was performed in triplicate with a QuantStudio^TM^ 6 Flex System (Applied Biosystems, Foster City, CA, USA) using 2 µL of cDNA template per 20 µL reaction containing 1× PowerUP^TM^ SYBER^®^ Green (Thermo Fisher Scientific, Waltham, MA, USA). The thermal program for the reactions was a hold period consisting of 2 min at 50 °C then 2 min at 95 °C, followed by 45 cycles of 95 °C for 15 s, 53.5 °C for 20 s, and 72 °C for 30 s. At the end of the PCR reaction, a melt-curve dissociation analysis was performed to confirm the product’s size. The region of DWV amplified was based on the following primers: Forward-GAG ATT GAA GCG CAT GAA CA; Reverse-TGA ATT CAG TGT CGC CCA TA ([49]; AY292384.1). DWV titers were determined using the standard curves of plasmid standards (generated by Invitrogen GeneArt Synthesis, Life Technologies Corporation, Carlsbad, CA, USA). Linearized plasmid standards, containing from 10^12^ to 10^5^ copies per reaction, were used as templates to assess primer efficiency and quantify the relative amount of virus following standard practices [50,51]. Linear standard equations were generated using the log10 of the initial plasmid copy number, and this was used to determine the DWV copy number. *Nosema* spores were counted following the standard protocol [52]. Worker bees (*n* = 25 per colony) were subsampled from a sample of about 300 bees collected from each of the 30 colonies two weeks after tagging (June 2015). For the amount of stored pollen, the technique used to estimate brood area as described by [53] was used. Stored pollen estimates were also conducted for the 30 colonies two weeks after all of the bee tagging and paint-marking had concluded in June 2015, September 2015, and March 2016.

### 2.6. Data Analyses

The following flight variables were analyzed: age of first flight, age at last flight (as a measure of longevity), total number of trips, trips per day, and total hours of foraging activities as a proxy for total distance traveled [39]. Prior to the analysis, all variables were subjected to square-root transformation to better approximate normality. In addition to pooling all of the data, a two-factor mixed model was also performed on each of the variables to compare the effects of honey bee stock and irradiation treatment. Analyses for all foraging flight parameters were performed on cohort groups to account for outside factors (i.e., weather) that would affect each group at different ages. Where interactions were found, slice tests were performed for each level of the interacting variables [54]. Before analyses of flight data, readings that occurred before 6:00 a.m. (sunrise) and after 8:00 p.m. (sunset) were excluded. Flights less than five minutes were also excluded to avoid orientation flights [35]. We further excluded outliers, such as flight activities that appeared to last over many hours as these were likely due to a read error [39]. Bees with a total ≤3 flights (probably due to tag loss, rejection by host bees, or early death), which were observed during their first 3 days, were also excluded from the analyses. Survival differences between the different treatments and stocks for RFID-tagged bees were analyzed using PROC LIFETEST [54].

The rejection of RFID-tagged bees has been a common experience for various studies that have used this technology. Of the 1503 bees that were RFID-tagged at the beginning of the project, a little under half (represented across 30 colonies) provided useful information throughout their lifetime for the statistical analyses. The distribution of tag loss (*n* = 386 bees; 26%), one-way reading (*n* =428 bees; 28%), and useful flight information (2-way readings) (*n* = 689 bees; 46%) (Table 1) was fairly equal across the treatment groups. At first, we experienced problems with interference among the readers, which may have caused one-directional data. In addition, one-directional readings were probably due to RFID-bees being dragged out of the host colony or unable to return when the bees took their orientation flights. It was also possible that some RFID-tagged bees were not detected by the readers, for which about a 30–50% detection success has been previously reported [45]. No detection of RIFD-tags may be due to bees leaving or entering through the tunnels sideways. Because of these reasons, the 2403 paint-marked bees were used to determine any negative effects of RFID tags on bee survival, and to confirm any effects noted in the RFID-tagged bees. Survival differences between the different treatments and stocks for paint-marked bees were also analyzed using PROC LIFETEST [54].

For the pathogen analyses, one-way analysis of variance was used to compare *Nosema* spore counts and the amount of stored pollen in June. Log-transformed DWV loads were analyzed via MANOVA (JMP v12.0) to examine the effect of comb irradiation on virus levels over time.

## 3. Results

### 3.1. Effects of Bee Genotype and Comb Irradiation on Age at Onset of Foraging

**Figure 1:**
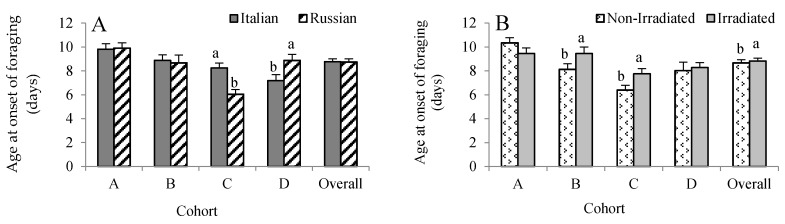
Effects of honey bee stock (**A**) and comb irradiation on age at onset of foraging (**B**) (mean ± standard error). For each cohort, bars with the same letters are not significantly different (*p* >0.05). In total, 340 RFID-tagged Russian and 349 Italian honey bees were analyzed (see Table 1).

All RFID-tagged bees were exposed to a uniform environment provided by a single host colony. Hence, we expected that all bees responded to the same needs of the host colony. There was no three-way interaction, but cohort × stock (*F* = 6.19, *p* = 0.0004) and cohort × treatment (*F* = 3.78, *p* = 0.011) influenced age at first foraging flight. Given this, the data were analyzed by cohorts. For Cohort A, no honey bee stock (*F* = 0.06, *p* = 0.801) and treatment (*F* = 2.05, *p* = 0.154) effects were detected (Figure 1A,B). For Cohort B, only comb irradiation influenced age at first foraging flight with the non-irradiated group foraging about 1.3 days earlier than the irradiated group. For Cohort C, we found that the Russian bees foraged 2.2 days earlier than the Italian bees (*F* = 20.12, *p* < 0.0001), while the non-irradiated group foraged 1.4 days earlier than the irradiated group (*F* = 6.66, *p* = 0.011). In contrast, the Italian bees in Cohort D foraged 1.7 days earlier than the Russian bees (*F* = 5.26, *p* = 0.024), while no irradiation effect was observed (*F* = 0.20, *p* = 0.652). Overall, there was no effect of honey bee stock on age at first foraging flight (*F* = 2.77, *p* = 0.097), but it was significantly affected by treatment, with those bees reared in non-irradiated combs foraging earlier than the bees reared in irradiated combs (*F* = 4.28, *p* = 0.039).

### 3.2. Effects of Bee Genotype and Comb Irradiation on Foraging Flights

**Figure 2:**
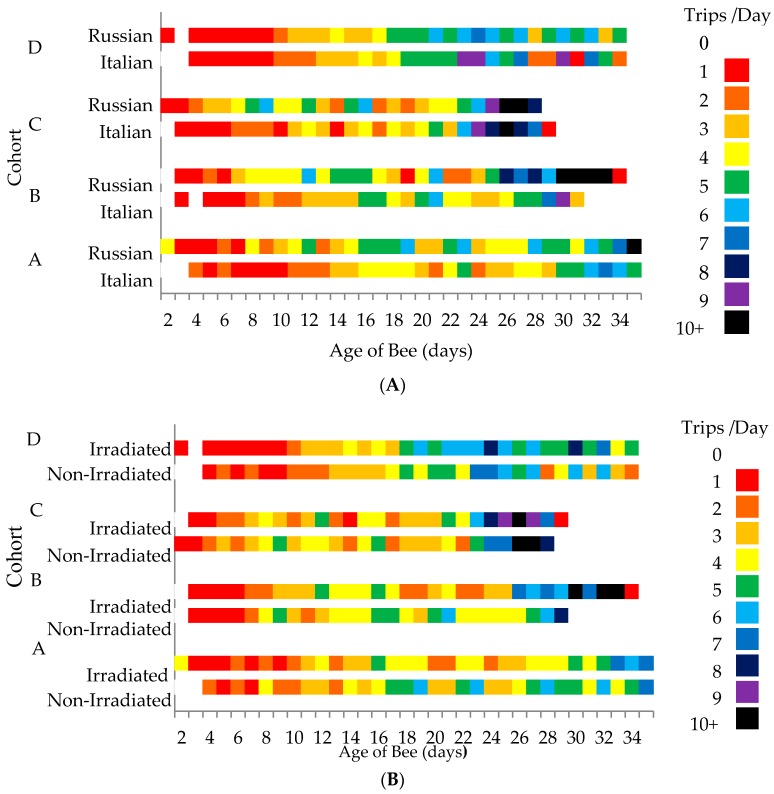
Number of daily flights of Russian and Italian honey bees (**A**) and bees that were reared from irradiated and non-irradiated combs (**B**) through time. Cohorts of bees were introduced into a host colony at different times.

Overall, only bee genotype affected the average number of daily flights (*F* = 16.77, *p* <0.0001), with the Russian honey bees conducting more foraging flights per day (3.83 ± 0.11 flights per day) than the Italian honey bees (3.25 ± 0.09 flights per day). In total, the Russian honey bees performed more foraging flights (31.33 ± 1.6 flights) than the Italian honey bees (25.35 ± 1.4 flights) throughout their lifetime (*F* = 7.30, *p* = 0.007). When daily foraging flights were analyzed by cohorts, the average number of daily flights was consistently higher in the Russian honey bees than the Italian honey bees, but significantly differed only in Cohorts A (*F* = 5.13, *p* = 0.025; Italian = 3.23 ± 0.14 flights per day, Russian = 3.85 ± 0.17 flights per day) and C (*F* = 6.30, *p* = 0.014; Italian = 3.14 ± 0.19 flights per day, Russian = 3.9 ± 0.24 flights per day). For both honey bee stocks, foraging activities gradually increased as the bees aged (Figure 2A). However, the Russian honey bees appeared to be more active by performing more flight activities through time than the Italian honey bees. When comparing the two treatment groups, those worker bees reared in non-irradiated combs performed more flights earlier than the bees reared in irradiated combs (Figure 2B).

**Figure 3:**
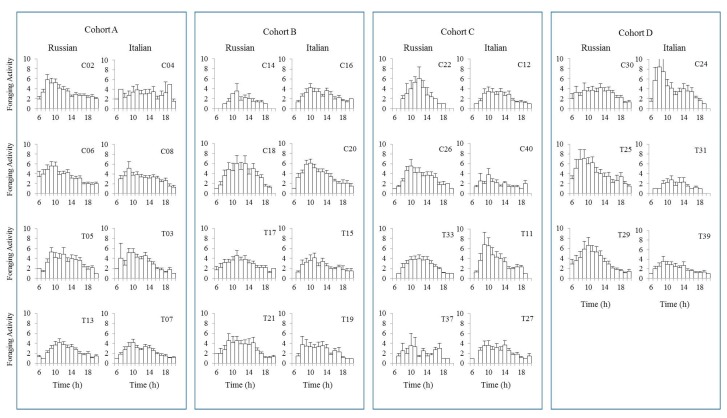
Daily flight patterns performed by Russian and Italian honey bee colonies throughout the experiment. Each bar represents an hour of the day starting from 6:00 a.m. (sunrise) to 8:00 p.m. (sunset).

Honey bees have the ability to synchronize the time of day to the time when flowers secrete nectar [55]. When we plot the level of flight activities for each of the test colonies, flight activities often began at sunrise (6:00 a.m.) and finished at sunset (8:00 p.m.) (Figure 3). However, some colonies started their flight activities 1–2 h later and also halted their activities 1–2 h earlier than other colonies. Overall, activity levels increased between 6:00 and 7:00 a.m., peaked before noon, and slowed thereafter. However, there were few colonies that showed steadily low levels of activities throughout the day for both honey bee stocks.

The variation in colony-level activities may be attributable to the differences in the activity levels of individual bees throughout their life span [45]. There were 15,003 flights analyzed in this study. When we looked at the top 25% of the total flight activities, the Russian honey bees contributed about 33% while the Italian honey bees accounted for 25%. Regarding comb treatment, bees reared in irradiated combs contributed 30.7% while those reared in non-irradiated combs did 27.2% of the total flights throughout the experiment.

### 3.3. Effects of Bee Genotype and Comb Irradiation on Flight Duration

**Figure 4:**
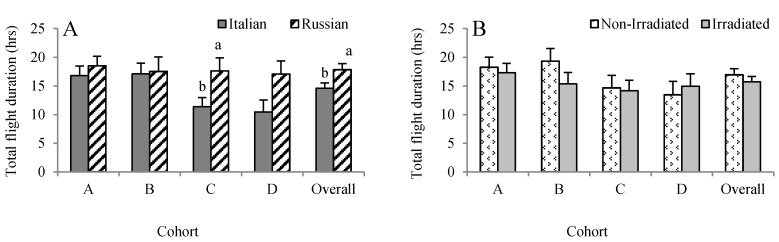
Effects of honey bee stock (**A**) and comb irradiation (**B**) on total duration of foraging flights (mean ± standard error). For each cohort, bars with the same letters are not significantly different (*p* > 0.05).

Overall, the duration of each individual flight lasted for about 31 min. Numerically, the Russian honey bees conducted longer flights than the Italian honey bees, but it was only significant in Cohort C (*F* = 5.75, *p* = 0.011) (Figure 4A). Although the Russian honey bees performed longer flights (1025 versus 627 min) than the Italian honey bees in Cohort D, the difference was not significant (*F* = 2.76, *p* = 0.0996). When all of the data were pooled, the flight duration was longer in Russian honey bees (1070.25 ± 63.44 min) than the Italian honey bees (876.50 ± 54.84 min) (*F* = 6.41, *p* = 0.012). Overall, irradiating combs did not influence the accumulated duration of flights the bees performed over their life span (*F* = 0.81, *p* = 0.369) (Figure 4B).

### 3.4. Effects of Bee Genotype and Comb Irradiation on Weight at Bee Emergence and Bee Survival

#### 3.4.1. Weight at Bee Emergence

For weight at bee emergence, we detected a significant interaction between honey bee stock and comb treatment (*F* = 25.5, *p* < 0.0001). In the Russian honey bees, those reared in non-irradiated combs were larger (112 ± 1.3 mg) than the bees reared in irradiated combs (105.9 ± 0.7 mg). In contrast, the Italian honey bees reared in the irradiated combs were heavier (110.6 ± 1.0 mg) than those from non-irradiated combs (107 ± 0.6 mg). Although previous work indicated that Russian honey bees are larger than Italian honey bees (94 versus 82 mg) [56], we did not detect any significant differences in weight between the two stocks (109 mg) (*F* = 0.01, *p* = 0.935) in this study. Overall, the newly emerged bees in this study were significantly larger than those reported by [56].

#### 3.4.2. Survival of RFID-Tagged Bees

For bee survival, a three-way interaction (cohort x honey bee stock x treatment) was detected (*F* = 6.02, *p* = 0.0005). For Cohort A, there was a significant interaction between honey bee stock and treatment (*F* = 5.07, *p* = 0.025), so a slice test was performed. In the Italian honey bees, no differences between irradiated (21.28 ± 0.88 days) and non-irradiated (20.17 ± 1.07 days) groups were detected (*F* = 0.48, *p* = 0.49), while the Russian honey bees that were reared in the non-irradiated combs (22.03 ± 0.70 days) lived longer than those raised in irradiated combs 18.98 ± 0.87 days) (*F* = 7.09, *p* = 0.008). For Cohort B, the Italian control (20.20 ± 0.76 days) lived longer than the irradiated group (16.16 ± 1.27 days) (*F* = 8.95, *p* = 0.003). In contrast, the Russian control group (16.97 ± 0.92 days) was shorter-lived than the Russian irradiated group 12.45 ± 1.88 days) (*F* = 7.29, *p* = 0.008). For Cohort C, no two-way interaction and no stock or treatment effects were detected. For Cohort D, only a stock effect was recorded, with the Russian honey bees (19.19 ± 0.91 days) surviving longer than the Italian honey bees (15.67 ± 0.94 days) (*F* = 6.33, *p* = 0.013). Overall, for both honey bee stocks, the RFID-tagged bees survived on average 18 days regardless of the comb treatment.

#### 3.4.3. Survival of Paint-Marked Bees

**Figure 5:**
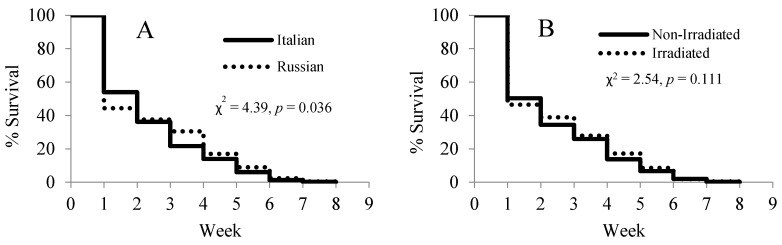
Effects of honey bee stock (**A**) and comb irradiation (**B**) on the survival of paint-marked bees. A total of 1411 Russian and 1292 Italian bees were marked.

A lifespan analysis of the paint-marked bees showed significant differences in the survival rates between the two honey bee stocks (χ^2^ = 4.39, *p* = 0.036) (Figure 5A), but no differences between the two comb treatments (χ^2^ = 2.54, *p* = 0.111) (Figure 5B). The Russian honey bees suffered from high loss in the first two weeks of the experiment, but had a slower decline than the Italian honey bees after the third week.

Comparing the survival of the paint-marked bees to the RFID-tagged bees, the bees seemed affected by RFID-tagging probably because of the stress of having additional load [18]. Some of the RFID-tagged bees were observed on the ground during their attempt to perform orientation flights. While some RFID-tagged bees were able to fly again, others were unable to get off the grass and presumably were preyed upon by ants. Some tagged bees were manually returned into the colonies. The last RFID-tagged bee survived until its 35th day (Figure 2), while the last paint-marked bee lasted for seven weeks (49 days). Although the additional stress brought by the weight of the tags on the bees may have negatively affected their survival [18], it should have been the same for both stocks.

### 3.5. Effects of Bee Genotype and Comb Irradiation on Viral Load, Nosema Spore Count, and Stored Pollen 

**Figure 6:**
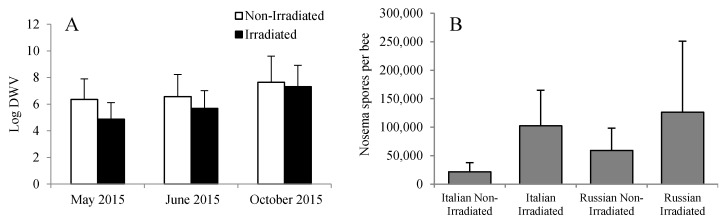
(**A**) Viral load and (**B**) *Nosema* spore count in Russian and Italian honey bee colonies containing irradiated or non-irradiated combs. For DWV, there was an overall significant reduction in viral load due to the irradiation treatment, with viral load increasing in general through time. DWV: *Deformed wing virus*.

Overall, the levels of DWV were significantly lower in bees that were reared in irradiated combs than in bees that were reared in non-irradiated combs (*F* = 4.09, *p* = 0.023). However, the difference was only evident early in the season (May and June) as compared to those that were reared six months post-irradiation treatment (Figure 6A). This discrepancy was probably due to the wide variation in DWV levels among the bees or colonies. It is possible that some of the bees analyzed were reared in previously infested cells or that the effectiveness of gamma irradiation is reduced through time. Numerically, the *Nosema* spore counts were higher in colonies with irradiated combs than in colonies with non-irradiated combs. However, no significant differences were detected (*F* = 0.26, *p* = 0.853) probably because of the wide variation among colonies (Figure 6B).

**Figure 7:**
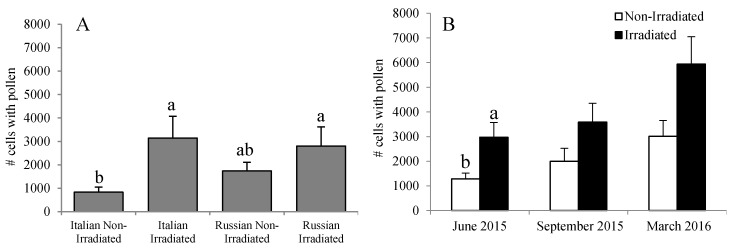
Amount of pollen stores in Russian and Italian honey bee colonies containing irradiated or non-irradiated combs (**A**) overall or (**B**) over the season. Bars with the same letters are not significantly different (*p* >0.05).

The amount of stored pollen mirrors the trend for *Nosema* spore counts. The highest pollen stores were recorded in the Russian and Italian colonies having irradiated combs, but were comparable to those of Russian colonies with non-irradiated combs in June (*F* = 3.17, *p* = 0.038) (Figure 7A). No effect of comb irradiation was observed in September 2015 (*F* = 3.14, *p* = 0.088) and March 2016 (*F* = 3.99, *p* = 0.066) (Figure 7B).

## 4. Discussion

Flight activity is an important component of foraging, which determines the overall performance or productivity of a colony [19]. Because of the inconsistencies and differences across cohorts of bees, this study showed that honey bee genotype and irradiation of combs where the bees were reared only had effects on some foraging parameters. These inconsistencies were probably due to the wide variation in the levels of foraging activities both at colony and individual levels. Experimental evidence has shown that the foraging patterns of European honey bees vary in differing environments [57]. In this study, all of our test bees representing 30 different colonies were exposed to the same environment provided by a single host colony. Yet, we found that bee genotype influenced the number of average daily flights, the total number of foraging flights, and total flight duration, in which the Russian bees outperformed the Italian bees. This observation suggests that Russian bees are probably more sensitive to even slight changes in colony status than Italian bees. Russian bees are known to be more responsive to pollen and nectar flows than Italian bees, by building up rapidly when resources are available and shutting down when resources disappear [58]. Differences in colony environment between the Italian and Russian bees when the test bees were developing may have also contributed to these discrepancies. Differential responses to colony environments had been observed in high- and low-pollen hoarding strain bees, which affected their development, behavior, and physical traits [37]. Despite having similar body mass (~109 mg), the Russian bees performed more flights, totaling to about 18 h of flight time throughout their lifetime, which was 3 h more than the Italian bees had. This observation suggests that Russian bees are probably more opportunistic or competitive in exploiting rich rewards than the Italian bees. It is also possible that the Russian bees may have a more efficient recruitment system or that they can better process information related to resources. This may help explain why honey production in Russian colonies is comparable if not better than in Italian colonies [59,60], despite their having generally smaller colony sizes [58,59]. Despite the high energy expenditures brought on by long flight durations in Russian bees, their longevity was similar to that of Italian bees (~18 days), which disagrees with the findings of [61] claiming that the survival of foragers is negatively associated with the duration of foraging activity or the number of foraging days. Bee genotype appears to influence this relationship.

Overall, exposing combs to gamma irradiation affected age at first flight, with the worker bees reared in non-irradiated combs foraging earlier than those reared in irradiated combs. However, this significant effect of comb irradiation was largely driven by two out of the four cohorts. Bees reared in non-irradiated combs foraged 1.33 days (Cohort B) and 1.37 days (Cohort C) earlier than those bees reared in irradiated combs. The premature foraging of bees reared in untreated combs may be influenced by the amount of pollen stores. It has been demonstrated that bees reared in colonies with low pollen stores forage earlier than those reared in colonies with high pollen stores [62]. While the Russian bees stored more pollen than the Italian bees on average, colonies that used gamma-irradiated combs stored the most pollen in both stocks of honey bees, even 11 months post-comb treatment. This ability of bees reared from irradiated combs to collect and store more pollen may be associated with the health status of the bees. In this study, we found lower levels of DWV in bees that were reared in the irradiated combs than bees reared in the non-irradiated combs. A recent study showed that DWV-injected bees foraged 2.3 days earlier than bees injected with control solution [18]. While the same authors reported an overall range of 12–17 days, our study only recorded an average of about 9 days for age at first flight. Differences in experimental methods, hive conditions, weather, geographical location, and the genotype of test bees may account for the discrepancy in our results. The exact source of DWV infecting our control bees is unknown. However, viruses have been detected in wax comb [63]. Thus, it is possible that the virus may have been acquired by the bees via the combs. Although all of the bees used in this study were uninfested with varroa mites, it is possible that some of the cells in the non-irradiated combs had previously supported the development of varroa-infested bees. Traces of DWV-infected mite feces may have been embedded in the wax or in between layers of cocoons, which possibly contaminated the larval food. CBPV has been detected in bee feces, and the virus extracted from the feces of infected bees has been shown to be infectious [12].

Other factors that affect the age of first foraging flight are *Nosema* infection [64] and the nutritional status of the hive [35]. *Nosema* infection causes precocious foraging [19,64], which is in disagreement with the findings of [39] claiming that infected bees were older when they started foraging. In this study, we did not count *Nosema* spores before the original bee population had turned over or from a subsample of our tagged bees. However, we detected varying but non-significant levels of *Nosema* infection of the resident bees. It is possible that some of our test bees had acquired *Nosema* during their larval development via horizontal transmission, which may have contributed to the inconsistent effects of comb irradiation on the age of first flight among the four cohorts of bees. A recent study has demonstrated that honey bee larvae can be infected by *N. ceranae* [65]. Further, *Nosema* causes atrophy of the hypopharyngeal glands, which is responsible for synthesizing and secreting royal jelly for developing bees [66]. Theoretically, some of our test bees may have had some developmental problems due to insufficient larval food. Nonetheless, *Nosema* infection does not cause energetic stress on *Nosema* tolerant bees, thus maintaining colony efficiency and productivity [67]. Genetic variation in resistance to *N. ceranae* has been documented in Russian bees, but not in the Italian lineage [68]. This observation may explain why the Russian bees were able to make longer flights despite the incidence of *Nosema* in the colonies.

Individual bees have been reported to adjust their foraging activities based on the needs of the colony [45]. However, variation among bees may also be associated with the health status of individual bees, since different pathogen levels may have different effects on foraging parameters or the endocrine system of bees. The energetic cost of viral infection, especially among honey bee stocks, needs to be assessed. In this study, we found that gamma irradiation of combs can lower the DWV levels of bees emerging from them, which may have contributed to the increase in pollen stores of colonies with irradiated combs. Nevertheless, the overall long-term effects of comb irradiation on colony performance have yet to be assessed. Since the varroa-resistant Russian honey bees accomplished more flights, resulting in longer total flight durations, our findings highlight the additional value of using resistant stocks to mitigate varroa mite problems, as their feces may also serve as a reservoir of DWV. However, the questions of whether or not mite feces are sources of viral infection and whether varroa-resistant bees are also resistant or tolerant to DWV remain to be resolved.

## 5. Conclusions 

Our data suggest that gamma irradiation of combs can help minimize the negative impact of DWV in honey bees. Since the colonies with irradiated combs stored more pollen than those with non-irradiated combs, crop pollination efficiency may be further improved when mite-resistant stocks are used, since they performed more flights and had longer flight durations.

## Figures and Tables

**Table 1:** Final number of Radio Frequency Identification (RFID)-tagged and paint-marked bees for all treatment groups. Numbers inside ( ) are the number of colonies tested.

Honey Bee Stock	Comb Treatment	RFID-Tagged Bees					# Paint-Marked Bees
		Cohort A	Cohort B	Cohort C	Cohort D	Total	
Italian	Non-irradiated	38 (2)	55 (2)	30 (2)	30 (1)	153 (7)	487
	Irradiated	78 (2)	33 (2)	43 (2)	42 (2)	196 (8)	646
Russian	Non-irradiated	76 (2)	16 (2)	30 (2)	29 (1)	151 (7)	584
	Irradiated	52 (2)	41 (2)	38 (2)	58 (2)	189 (8)	686
TOTAL		244 (8)	145 (8)	141 (8)	159 (6)	689 (30)	2403

## References

[1] VanEngelsdorp D., Meixner M.D. (2010). A historical review of managed honey bee populations in europe and the united states and the factors that may affect them. J. Invertebr. Pathol..

[2] Kralj J., Brockmann A., Fuchs S., Tautz J. (2007). The parasitic mite varroa destructor affects non-associative learning in honey bee foragers, *Apis mellifera* L.. J. Comp. Physiol. A.

[3] Kralj J., Fuchs S. (2004). Parasite-Host Interactions between Varroa Destructor Anderson and Trueman and Apis Mellifera L.: Influence of Parasitism on Flight Behaviour and on the Loss of Infested Foragers.

[4] Kralj J., Fuchs S. (2006). Parasitic varroa destructor mites influence flight duration and homing ability of infested apis mellifera foragers. Apidologie.

[5] De Miranda J.R., Genersch E. (2010). Deformed wing virus. J. Invertebr. Pathol..

[6] Ryabov E.V., Fannon J.M., Moore J.D., Wood G.R., Evans D.J. (2016). The iflaviruses sacbrood virus and deformed wing virus evoke different transcriptional responses in the honeybee which may facilitate their horizontal or vertical transmission. PeerJ.

[7] Möckel N., Gisder S., Genersch E. (2011). Horizontal transmission of deformed wing virus: Pathological consequences in adult bees (*Apis mellifera*) depend on the transmission route. J. Gen. Virol..

[8] Katznelson H., Jamieson C., Lawton E., Bellamy W. (1952). Studies on the treatment of contaminated combs and honey with high velocity electrons. Can. J. Technol..

[9] Hansen H., Brødsgaard C.J. (1999). American foulbrood: A review of its biology, diagnosis and control. Bee World.

[10] Gilliam M., Prest D.B. (1987). Microbiology of feces of the larval honey bee, apis mellifera. J. Invertebr. Pathol..

[11] Bailey L. (1953). The transmission of nosema disease. Bee World.

[12] Ribière M., Lallemand P., Iscache A.-L., Schurr F., Celle O., Blanchard P., Olivier V., Faucon J.-P. (2007). Spread of infectious chronic bee paralysis virus by honeybee (*Apis mellifera* L.) feces. Appl. Environ. Microbiol..

[13] Donzé G., Herrmann M., Bachofen B., Guerin P.R.M. (1996). Effect of mating frequency and brood cell infestation rate on the reproductive success of the honeybee parasite varroa jacobsoni. Ecol. Entomol..

[14] Kralj J., Fuchs S. (2010). *Nosema* sp. Influences flight behavior of infected honey bee (*Apis mellifera*) foragers. Apidologie.

[15] Mayack C., Naug D. (2009). Energetic stress in the honeybee apis mellifera from nosema ceranae infection. J. Invertebr. Pathol..

[16] Li Z., Chen Y., Zhang S., Chen S., Li W., Yan L., Shi L., Wu L., Sohr A., Su S. (2013). Viral infection affects sucrose responsiveness and homing ability of forager honey bees, *Apis mellifea* L.. PLoS ONE.

[17] Dussaubat C., Maisonnasse A., Crauser D., Beslay D., Costagliola G., Soubeyrand S., Kretzchmar A., Le Conte Y. (2013). Flight behavior and pheromone changes associated to nosema ceranae infection of honey bee workers (*Apis mellifera*) in field conditions. J. Invertebr. Pathol..

[18] Benaets K., Van Geystelen A., Cardoen D., De Smet L., de Graaf D.C., Schoofs L., Larmuseau M.H.D., Brettell L.E., Martin S.J., Wenseleers T. (2017). Covert deformed wing virus infections have long-term deleterious effects on honeybee foraging and survival. Proc. R. Soc. B Biol. Sci..

[19] Dosselli R., Grassl J., Carson A., Simmons L.W., Baer B. (2016). Flight behaviour of honey bee (*Apis mellifera*) workers is altered by initial infections of the fungal parasite nosema apis. Sci. Rep..

[20] Hallman G.J. (2013). Control of stored product pests by ionizing radiation. J. Stored Prod. Res..

[21] Kume T., Furuta M., Todoriki S., Uenoyama N., Kobayashi Y. (2009). Status of food irradiation in the world. Radiat. Phys. Chem..

[22] Hornitzky M.A.Z. (1994). Commercial use of gamma radiation in the beekeeping industry. Bee World.

[23] Gochnauer T.A., Hamilton H.A. (1970). Disinfection of honeybee combs by gamma irradiation I. American foul brood disease. J. Apic. Res..

[24] Gosselin P., Charbonneau R. (1990). Disinfection of the bee hive’s american foulbrood by gamma radiation from cobalt-60. Int. J. Radiat. Appl. Instrum. Part C Radiat. Phys. Chem..

[25] Baggio A., Gallina A., Dainese N., Manzinello C., Mutinelli F., Serra G., Colombo R., Carpana E., Sabatini A.G., Wallner K. (2005). Gamma radiation: A sanitating treatment of afb-contaminated beekeeping eqiupment. Apiacta.

[26] De Guzman Z.M., Cervancia C.R., Dimasuay K.G.B., Tolentino M.M., Abrera G.B., Cobar M.L.C., Fajardo A.C., Sabino N.G., Manila-Fajardo A.C., Feliciano C.P. (2011). Radiation inactivation of *Paenibacillus larvae* and sterilization of american foul brood (afb) infected hives using co-60 gamma rays. Appl. Radiat. Isot..

[27] Hume A., Ames J., Rennick L., Duprex W., Marzi A., Tonkiss J., Mühlberger E. (2016). Inactivation of rna viruses by gamma irradiation: A study on mitigating factors. Viruses.

[28] Thomas F.C., Davies A.G., Dulac G.C., Willis N.G., Papp-Vid G., Girard A. (1981). Gamma ray inactivation of some animal viruses. Can. J. Comp. Med..

[29] Katznelson H., Robb J. (1962). The use of gamma radiation from cobalt-60 in the control of diseases of the honeybee and the sterilization of honey. Can. J. Microbiol..

[30] Pankiw P., Bailey L., Gochnauer T.A., Hamilton H.A. (1970). Disinfection of honeybee combs by gamma irradiation. II. European foul brood disease. J. Apic. Res..

[31] Spivak M., Reuter G.S. (2001). Resistance to american foulbrood disease by honey bee colonies *Apis mellifera* bred for hygienic behavior. Apidologie.

[32] Rinderer T.E., Harris J.W., Hunt G., de Guzman L.I. (2010). Breeding for resistance to *Varroa destructor* in north america. Apidologie.

[33] Büchler R., Berg S., Le Conte Y. (2010). Breeding for resistance to *Varroa destructor* in europe. Apidologie.

[34] Invernizzi C., Rivas F., Bettucci L. (2011). Resistance to chalkbrood disease in apis mellifera l. (hymenoptera: Apidae) colonies with different hygienic behaviour. Neotrop. Entomol..

[35] Winston M.L. (1987). The Biology of the Honey Bee.

[36] Guzman-Novoa E., Gary N.E. (1993). Genotypic variability of components of foraging behavior in honey bees (hymenoptera: Apidae). J. Econ. Entomol..

[37] Pankiw T., Tarpy D.R., Page R.E. (2002). Genotype and rearing environment affect honeybee perception and foraging behaviour. Anim. Behav..

[38] Scheiner R., Page R.E., Erber J. (2001). Responsiveness to sucrose affects tactile and olfactory learning in preforaging honey bees of two genetic strains. Behav. Brain Res..

[39] Lach L., Kratz M., Baer B. (2015). Parasitized honey bees are less likely to forage and carry less pollen. J. Invertebr. Pathol..

[40] Rinderer T.E., de Guzman L.I., Delatte G.T., Stelzer J.A., Kuznetsov V.N., Beaman L.D., Watts R., Harris J. (2001). Resistance to the parasitic mite *Varroa destructor* in honey bees from far-eastern russia. Apidologie.

[41] Rinderer T.E., de Guzman L.I., Danka R. (2005). A new phase begins for the usda-ars russian honey bee breeding program. Am. Bee J..

[42] Stelzer R.J., Chittka L. (2010). Bumblebee foraging rhythms under the midnight sun measured with radiofrequency identification. BMC Biol..

[43] Robinson E.J.H., Feinerman O., Franks N.R. (2009). Flexible task allocation and the organization of work in ants. Proc. R. Soc. B Biol. Sci..

[44] Decourtye A., Devillers J., Aupinel P., Brun F., Bagnis C., Fourrier J., Gauthier M. (2011). Honeybee tracking with microchips: A new methodology to measure the effects of pesticides. Ecotoxicology.

[45] Tenczar P., Lutz C.C., Rao V.D., Goldenfeld N., Robinson G.E. (2014). Automated monitoring reveals extreme interindividual variation and plasticity in honeybee foraging activity levels. Anim. Behav..

[46] Schneider C.W., Tautz J., Grünewald B., Fuchs S. (2012). Rfid tracking of sublethal effects of two neonicotinoid insecticides on the foraging behavior of apis mellifera. PLoS ONE.

[47] Thompson H., Coulson M., Ruddle N., Wilkins S., Harkin S. (2016). Thiamethoxam: Assessing flight activity of honeybees foraging on treated oilseed rape usinf radio frequency identification technology. Environ. Toxicol. Chem..

[48] Pankiw T., Page R.E. (2001). Genotype and colony environment affect honeybee (*Apis mellifera* L.) development and foraging behavior. Behav. Ecol. Sociobiol..

[49] Boncristiani H., Underwood R., Schwarz R., Evans J.D., Pettis J., vanEngelsdorp D. (2012). Direct effect of acaricides on pathogen loads and gene expression levels in honey bees *Apis mellifera*. J. Insect Physiol..

[50] Hou Y., Zhang H., Miranda L., Lin S. (2010). Serious overestimation in quantitative pcr by circular (supercoiled) plasmid standard: Microalgal pcna as the model gene. PLoS ONE.

[51] Cavigli I., Daughenbaugh K.F., Martin M., Lerch M., Banner K., Garcia E., Brutscher L.M., Flenniken M.L. (2016). Pathogen prevalence and abundance in honey bee colonies involved in almond pollination. Apidologie.

[52] Fries I., Chauzat M.-P., Chen Y.-P., Doublet V., Genersch E., Gisder S., Higes M., McMahon D.P., Martín-Hernández R., Natsopoulou M. (2013). Standard methods for nosema research. J. Apic. Res..

[53] Rogers L.E., Gilbert R.O., Burgett M. (1983). Sampling honeybee colonies for brood production: A double sampling technique. J. Apic. Res..

[54] SAS Institute (1992). Sas Technical Report P-229, Sas/Stat Software: Changes and Enhancements, Release 6.07.

[55] Beling I. (1929). Über das zeitgedächtnis der bienen. Z. Vgl. Physiol..

[56] Rinkevich F.D., Margotta J.W., Pittman J.M., Danka R.G., Tarver M.R., Ottea J.A., Healy K.B. (2015). Genetics, synergists, and age affect insecticide sensitivity of the honey bee, apis mellifera. PLoS ONE.

[57] Waddington K.D., Herbert T.J., Visscher P.K., Richter M.R. (1994). Comparisons of forager distributions from matched honey bee colonies in suburban environments. Behav. Ecol. Sociobiol..

[58] Tubbs H., Harper C., Bigalk M., Bernard S.J., Delatte G.T., Sylvester H.A., Rinderer T.E. (2003). Commercial management of ars russian honey bees. Am. Bee J..

[59] Rinderer T.E., de Guzman L.I., Harper C. (2004). The effects of co-mingled russian and italian honey bee stocks and sunny or shaded apiaries on varroa mite infestation level, worker bee population and honey production. Am. Bee J..

[60] Rinderer T.E., de Guzman L.I., Delatte G.T., Stelzer J.A., Lancaster V.A., Williams J.L., Beaman L.D., Kuznetsov V., Bigalk M., Bernard S.J. (2001). Multi-state field trials of ars russian honey bees 2. Honey production 1999, 2000. Am. Bee J..

[61] Visscher P.K., Dukas R. (1997). Survivorship of foraging honey bees. Insectes Sociaux.

[62] Janmaat A.F., Winston M.L. (2000). The influence of pollen storage area and varroa jacobsoni oudemans parasitism on temporal caste structure in honey bees (*Apis mellifera* L.). Insectes Sociaux.

[63] Colwell M.J., Currie R.W., Pernal S.F., Simone-Finstrom M. (2017). Viruses in unexpected places: New transmission routes of european honey bee (*Apis mellifera*) viruses. Proceedings of the American Bee Research Conference.

[64] Goblirsch M., Huang Z.Y., Spivak M. (2013). Physiological and behavioral changes in honey bees (*Apis mellifera*) induced by nosema ceranae infection. PLoS ONE.

[65] Eiri D.M., Suwannapong G., Endler M., Nieh J.C. (2015). Nosema ceranae can infect honey bee larvae and reduces subsequent adult longevity. PLoS ONE.

[66] Ptaszyńska A.A., Borsuk G., Anusiewicz M., Mułenko W. (2012). Location of *Nosema* spp. Spores within the body of the honey bee. Med. Weter..

[67] Kurze C., Mayack C., Hirche F., Stangl G.I., Le Conte Y., Kryger P., Moritz R.F.A. (2016). *Nosema* spp. Infections cause no energetic stress in tolerant honeybees. Parasitol. Res..

[68] Bourgeois A.L., Rinderer T.E., Sylvester H.A., Holloway B., Oldroyd B.P. (2012). Patterns of *Apis mellifera* infestation by nosema ceranae support the parasite hypothesis for the evolution of extreme polyandry in eusocial insects. Apidologie.

